# Aging and Advanced Dementia Matters: End-of-Life Care in Nursing Homes

**DOI:** 10.1093/geroni/igaa057.1679

**Published:** 2020-12-16

**Authors:** Ruth Lopez, Ellen McCarthy, Meghan Hendricksen, Susan McLennon, Anita Rogers, LaKeva Harris, Ashley Roach, Susan Mitchell

**Affiliations:** 1 University of Tennessee Knoxville, Knoxville, Tennessee, United States; 2 Hebrew SeniorLife, Harvard Medical School, roslindale, Massachusetts, United States; 3 Hebrew SeniorLife, Roslindale, Minnesota, United States; 4 University of Tennessee-Knoxville, KNOXVILLE, Tennessee, United States; 5 University of Tennessee at Martin, Parsons, Tennessee, United States; 6 University of Tennessee, Portland, Oregon, United States; 7 Marcus Institute for Aging Research, Roslindale, Massachusetts, United States

## Abstract

Over 5 million Americans have dementia, and the majority will die in nursing home (NHs). While comfort is the main goal of care for most NH residents with advanced dementia, they commonly receive burdensome and costly interventions such as hospital transfers and feeding tubes that are of little clinical benefit. Despite 20 years of research and numerous experts and associations advocating a palliative approach to care, quantitative studies continue to demonstrate striking and persistent regional, facility, and racial differences, including: greater intensity care among African American versus White residents; greater intensity of care in the Southeastern US; and wide variation in care among NHs in the same region of the country. The reasons for these differences are poorly understood. Assessment of Disparities and Variation for Alzheimer’s disease in Nursing home Care at End of life (ADVANCE) is a 3-year, NIA funded qualitative study of 16 NHs in 4 regions of the country which aims to explain regional and racial factors influencing feeding tube and hospital transfer rates. The purpose of this presentation is to present the methodology established in this study and to highlight factors challenging and enabling implementation of the study protocol. To date, data have been collected in 11 NHs, and include 135 staff interviews, 40 proxy interviews, and nearly 800 hours of observation. These findings demonstrate that although challenging, large qualitative research is possible and holds promise as an effective method to illuminate complex processes influencing end-of-life care for NH residents with advanced dementia.

